# Resting State Brain Networks under Inverse Agonist versus Complete Knockout of the Cannabinoid Receptor 1

**DOI:** 10.1021/acschemneuro.3c00804

**Published:** 2024-04-04

**Authors:** Hui Li, Qiong Ye, Da Wang, Bowen Shi, Wenjing Xu, Shuning Zhang, Xiaoyang Han, Xiao-Yong Zhang, Garth J. Thompson

**Affiliations:** † iHuman Institute, ShanghaiTech University, Shanghai 201210, China; ‡ High Magnetic Field Laboratory, Hefei Institutes of Physical Science, Chinese Academy of Sciences, Hefei, Anhui 230031, China; § School of Life Science and Technology, ShanghaiTech University, Shanghai 201210, China; ∥ Institute of Science and Technology for Brain-Inspired Intelligence, Fudan University, Shanghai 200433, China; ⊥ Key Laboratory of Computational Neuroscience and Brain-Inspired Intelligence, Fudan University, Ministry of Education, Shanghai 200433, China

**Keywords:** cannabinoid receptor 1, gene knockout, rimonabant, magnetic resonance imaging, network activity, directional uniformity

## Abstract

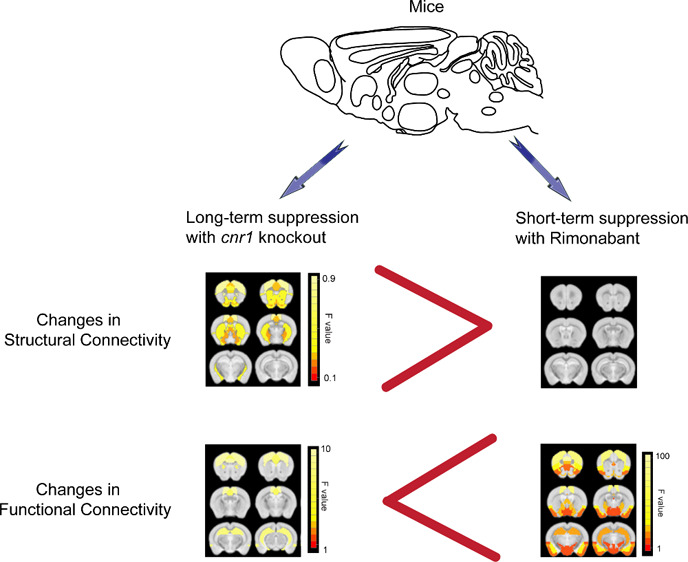

The cannabinoid receptor 1 (CB_1_) is famous as the target of Δ^9^-tetrahydrocannabinol (THC), which is the active ingredient of marijuana. Suppression of CB_1_ is frequently suggested as a drug target or gene therapy for many conditions (e.g., obesity, Parkinson’s disease). However, brain networks affected by CB_1_ remain elusive, and unanticipated psychological effects in a clinical trial had dire consequences. To better understand the whole brain effects of CB_1_ suppression we performed in vivo imaging on mice under complete knockout of the gene for CB_1_ (*cnr1*^*–/–*^) and also under the CB_1_ inverse agonist rimonabant. We examined white matter structural changes and brain function (network activity and directional uniformity) in *cnr1*^*–/–*^ mice. In *cnr1*^*–/–*^ mice, white matter (in both sexes) and functional directional uniformity (in male mice) were altered across the brain but network activity was largely unaltered. Conversely, under rimonabant, functional directional uniformity was not altered but network activity was altered in cortical regions, primarily in networks known to be altered by THC (e.g., neocortex, hippocampal formation). However, rimonabant did not alter many brain regions found in both our *cnr1*^*–/–*^ results and previous behavioral studies of *cnr1*^*–/–*^ mice (e.g., thalamus, infralimbic area). This suggests that chronic loss of *cnr1* is substantially different from short-term suppression, subtly rewiring the brain but largely maintaining the network activity. Our results help explain why pathological mutations in CB_1_ (e.g., chronic pain) do not always provide insight into the side effects of CB_1_ suppression (e.g., clinical depression), and thus urge more preclinical studies for any drugs that suppress CB_1_.

## Introduction

The cannabinoid receptor 1, CB_1_, is the target of Δ^9^-tetrahydrocannabinol (THC), the psychoactive ingredient of marijuana. CB_1_ is of great interest to medicine and biology, recently in part due to the legalization or decriminalization of marijuana across parts of North America and Europe. CB_1_ is distributed broadly throughout the central nervous system^1−3^ and human genetic variants of CB_1_ are linked to many psychiatric disorders (substance dependence, eating disorders, schizophrenia,^4^ pain sensitivity, sleep and memory disorders),^5^ and altered CB_1_ signaling is observed in other disorders (alcohol use disorder, schizophrenia, post-traumatic stress disorder, eating disorders,^6^ and Parkinson’s disease).^7^ For this reason, the agonism or antagonism of CB_1_ is a key therapeutic target for drug or gene therapy development. In particular, antagonism of the CB_1_ receptor has been suggested since 2002 as a potential target for Parkinson’s disease treatment,^8,9^ and since 2003 as a potential target for reducing obesity.^10−12^

While the endogenous spatial distribution of CB_1_ in the brain is well-known,^13,14^ CB_1_’s endogenous function in terms of brain networks has proven more elusive as most previous studies have focused on limited brain regions, rather than brain-spanning networks.^15−17^ Particularly, the extensive distribution of CB_1_ and ubiquity of endogenous cannabinoids (particularly anandamide) suggest that systemic agonism or antagonism has a high risk of side effects due to impact on multiple brain networks. Focusing on antagonism, in a mouse model of Parkinson’s disease, antagonism of CB_1_ with AM251 improved factors such as balance and muscle strength but increased anxious- and depressive-like behaviors. Antagonism was also considered as a treatment for obesity. The drug rimonabant, an inverse agonist of CB_1_ with high binding affinity, initially showed excellent results in reducing weight in rodents.^18^ However, clinical trials proved disastrous as patients reported adverse psychiatric effects, and the trials were ended when two patients committed suicide.^19^ A better and more systemic understanding of the brain networks involved with CB_1_ is, thus, greatly needed.

To investigate the systemic effects of a receptor, such as CB_1_, across the whole brain, transgenic animal lines are a key tool. Complete knockout mice as compared to wild-type (WT) mice are a common and essential first step for clues about what a gene “normally does”.^20^ Magnetic resonance imaging (MRI) can image the entire brain concurrently and thus is a key technique for understanding large brain networks. We thus combined these techniques by imaging wild-type (WT) mice versus mice with complete knockout of *cnr1*, the gene for CB_1_ (*cnr1*^*–/–*^) with high-field MRI (11.7 T). This can provide a baseline of networks endogenously affected by CB_1_ in the brain. These results can then be compared to the alteration of brain network activity measured by MRI under the drug rimonabant. In this manner, long-term removal of CB_1_ can be compared to short-term suppression of CB_1_ via rimonabant, and thus, long-term and short-term effects are compared across many brain regions.

Brain regions were examined in terms of structure and function and compared between the groups. We initially focused on white matter structure, as our previous work found molecular differences in axons.^21^ Diffusion tensor imaging (DTI)^22−24^ was used to examine the structural directional uniformity of brain communication. However, DTI can observe only long-term changes to the structure of white matter. Thus, to measure brain function, two functional MRI (fMRI) methods were used. First, resting state fMRI (rs-fMRI)^25^ was used to measure brain network activity. Second, spatio-temporal correlation tensors (STCT)^26,27^ were used to measure the functional directional uniformity of brain communication in white matter. The fMRI methods were applied to both long-term (*cnr1* knockout^*-*^) and short-term (rimonabant) changes.

Studies of the effects of THC^28^ and brain regions involved in behavioral changes in *cnr1*^–/–^ mice^16,29,30^ provide a list of hypothetical brain regions we expect to see directional uniformity or network activity altered within. Thus, we hypothesize regions rich in CB_1_ (e.g., neocortex and hippocampal formation) and also altered behavior-linked regions (e.g., thalamus and infralimbic area) will be altered. Furthermore, we hypothesize that there is substantial overlap between brain regions affected by both short-term and long-term suppression of CB_1_. However, regions we find that do not overlap between short-term and long-term suppression of CB_1_ may be of particular interest to future studies. This is because such regions may indicate limits regarding how many mutations in CB_1_ are able to help us understand the effects of exogenous drugs.

## Results

Brain region name abbreviations are given in Table S1.

### Diffusion Tensor Imaging (DTI) in *cnr1*^*–/–*^

**Figure 1:**
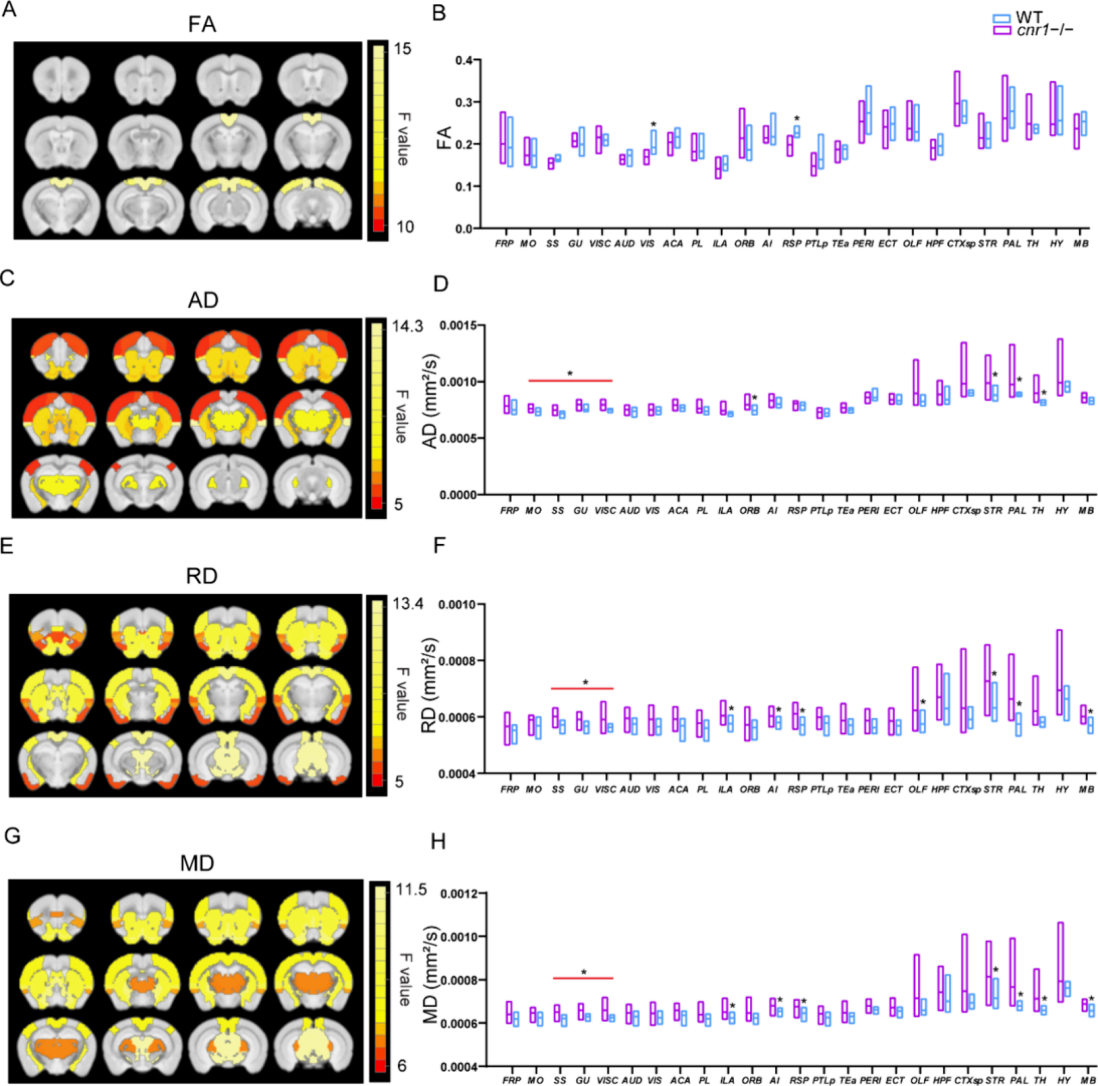
Comparison of diffusion tensor imaging (DTI) parameters between *cnr1*^*–/–*^ mice and WT mice. Significant brain regions (ANOVA genotype factor, *p* < 0.05, SGoF corrected) have been colored based on that region’s *F* value and superimposed over the T2 template image. Twelve slices are shown from rostral to caudal, top of brain upward. Results are shown for fractional anisotropy (FA) (A), axial diffusivity (AD) (C), radial diffusivity (RD) (E), and mean diffusivity (MD) (G). Group comparison of FA (B), AD (D), RD (F), and RD (H) in individual brain regions. Data are mean ± s.e.m. (*N* = 12 for *cnr1*^*–/–*^ and *N* = 10 for WT). * indicates *p* ≤ 0.05 corrected with SGoF. Regions of interest names are in Table S1.

To begin the investigation into the effects of CB_1_ on large-scale brain function, because our previous work demonstrated that the microstructure of white matter differed between WT and *cnr1*^*–/–*^,^21^ we first focused on white-matter structure changes, measured with DTI-derived metrics. Fractional anisotropy (FA) is a commonly used parameter in DTI that measures how directed (anisotropic) each voxel is. It has been shown to be a fairly nonspecific biomarker of microstructural architecture and neuropathology.^22^ Compared to WT mice, *cnr1*^–/–^ mice had significantly higher FA values in visual areas (VIS) and the retrosplenial area (RSP) (ANOVA genotype factor, SGoF corrected *p* < 0.05, sex and interaction factors not significant) (Figure 1A, B, Table 1).

**Table 1:**
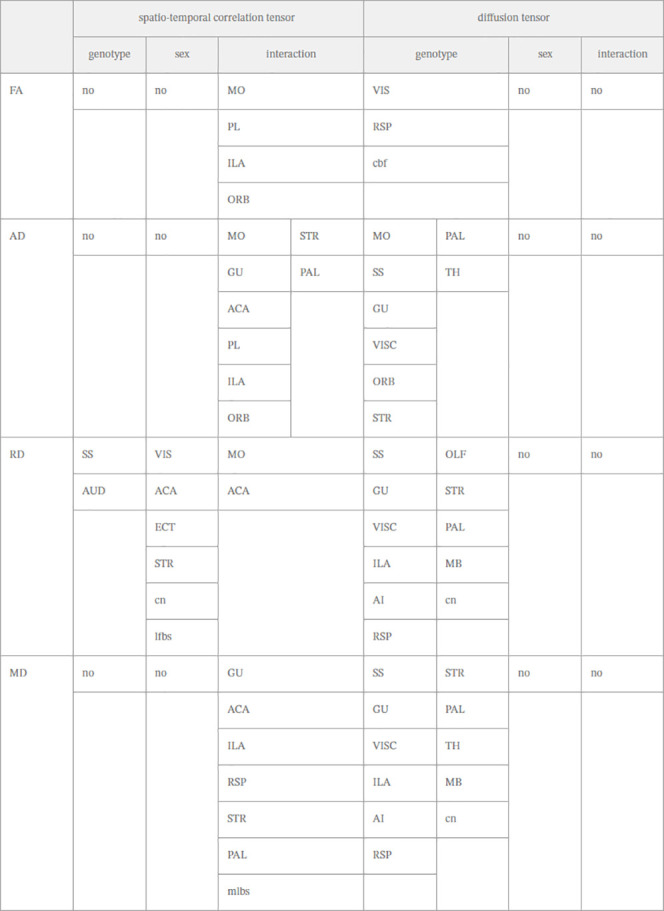
Significant Brain Regions Resulting from Two-Way ANOVA with Interaction of FA, AD, RD, and MD in STCT and DTI metrics^a^

a “Interaction” is the interaction factor between sex and genotype. *N* = 24 for STCT (6 male WT, 6 female WT, 6 male *cnr1*^–/–^, 6 female *cnr1*^–/–^), *N* = 22 for DTI (5 male WT, 5 female WT, 6 male *cnr1*^–/–^, 6 female *cnr1*^–/–^). The significant brain regions (inter-ROI level SGoF corrected *p* < 0.05) are listed in the table for the genotype, sex, and interaction factors for which they were statistically significant.

As FA is a summary measure of microstructural changes, greater specificity regarding the neurobiological changes can be further characterized by radial diffusivity (RD), axial diffusivity (AD), and mean diffusivity (MD).^24^ AD measures diffusion parallel to the axonal fibers, and has been associated with the integrity of axons.^24^ Compared to that in WT mice, AD was significantly higher in the following brain regions of *cnr1*^–/–^ mice: MO, somatosensory area (SS), gustatory area (GU), visceral area (VISC), ORB, striatum (STR), pallidum (PAL), and thalamus (TH) (ANOVA genotype factor, SGoF corrected *p* < 0.05, sex and interaction factors not significant) (Figure 1C, D, Table 1).

RD measures diffusion orthogonal to the direction of fibers, and it is an indirect indicator of myelin injury and decreased myelination.^23,24^ Compared to WT mice, RD also increased in the majority of brain regions of *cnr1*^–/–^ mice, with significant increases in the SS, GU, VISC, ILA, AI, RSP, OLF, STR, PAL, and midbrain (MB) (ANOVA genotype factor, SGoF corrected *p* < 0.05, sex and interaction factors not significant) (Figure 1E, F, Table 1).

We also calculated the mean diffusivity (MD). MD can be used to quantify membrane density and is sensitive to cell structure, edema, or necrosis. MD showed a trend similar to that of RD, significantly in the following brain areas: SS, GU, VISC, ILA, AI, RSP, OLF, STR, PAL, and TH (ANOVA genotype factor, SGoF corrected *p* < 0.05, sex and interaction factors not significant) (Figure 1G, H, Table 1).

### Resting State Functional MRI (rs-fMRI) in *cnr1*^*–/–*^

rs-fMRI was used to investigate the difference in brain function between *cnr1*^–/–^ and WT mice. Using rs-fMRI data, we calculated the correlation coefficients with seeds located in 25 gray matter brain regions to examine changes in standard region-to-region functional connectivity.^31^ Although the correlation coefficients between the 25 areas were lower on average in *cnr1*^–/–^ mice than in WT mice (Figure S2), there was no statistically significant difference (ANOVA, genotype, sex, and interaction factors not significant).

### Per-Voxel rs-fMRI Metrics in *cnr1*^*–/–*^

**Figure 2:**
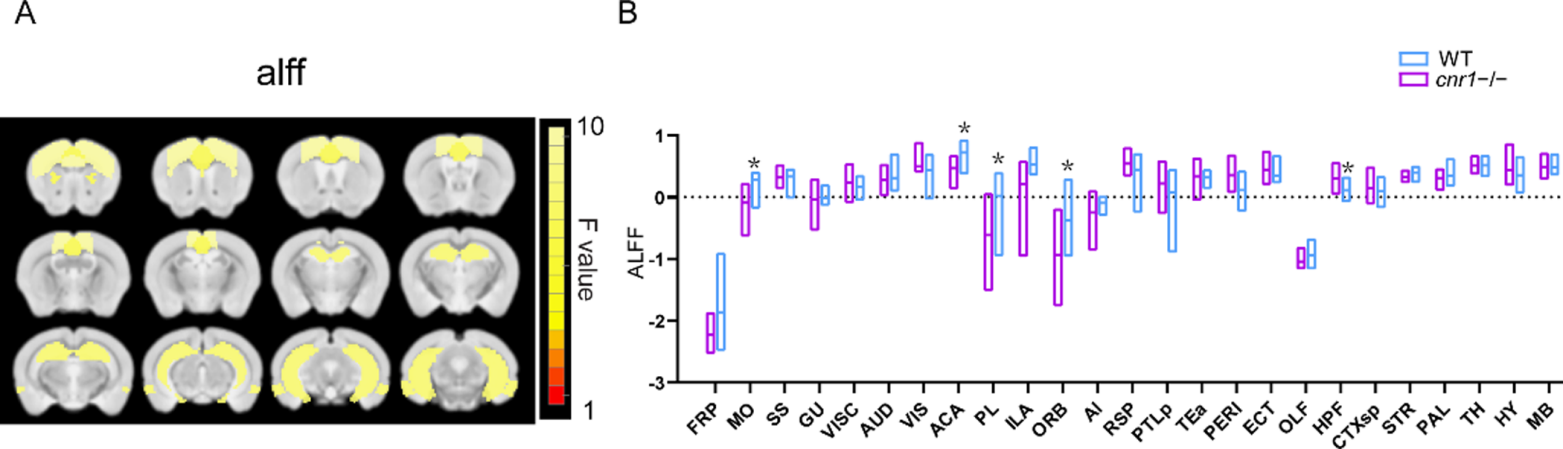
Comparison of the amplitude of low-frequency fluctuations (ALFF) between *cnr1*^–/–^ mice and WT mice. (A) Significant brain regions (ANOVA genotype factor, *p* < 0.05, SGoF corrected) have been colored based on that region’s *F* value and superimposed over the T2 template image. Twelve slices are shown from rostral to caudal, top of brain upward. (B) Group comparison of ALFF. Data are mean ± s.e.m (*N* = 10 for *cnr1*^–/–^ and *N* = 10 for WT). * Indicates *p* ≤ 0.05 corrected with SGoF. Region of interest names are in Table S1.

The amplitude of low-frequency fluctuations (ALFF)^32^ and functional connectivity density (FCD)^33^ are two different fMRI methods that reflect voxel-level local neural activity and voxel-level functional integration, respectively. By averaging all voxels within a given brain region, aspects of functional connectivity within that individual brain region can be quantified.^25^ Thus, we calculated ALFF, global FCD (gFCD), and local FCD (lFCD) maps to evaluate region-specific changes in functional connectivity. The hippocampus (HPF) of the *cnr1*^–/–^ mice had significantly larger ALFF than that of the WT group, while the somatomotor area (MO), anterior cingulate area (ACA), prelimbic area (PL), and orbital area (ORB) had significantly smaller ALFF than that of the WT group (ANOVA genotype factor, SGoF corrected *p* < 0.05, sex and interaction factors not significant) (Figure 2).

However, there were no statistically significant (or even discernible visible trend in) differences in either lFCD or gFCD between *cnr1*^*–/–*^ and WT mice (ANOVA, genotype, sex, and interaction factors not significant) (Figure S3).

### Spatio-temporal Correlation Tensor (STCT) in *cnr1*^*–/–*^

In addition to direction-insensitive functional connectivity, correlations in small amplitude fluctuations between a voxel and its close neighbors can be used to determine the direction of synchronous variation in the local neighborhoods around each voxel. We used the method described by Ding et al.^34,35^ to capture these as tensors and examine white matter using fMRI data. We calculated the STCT-derived parameters between *cnr1*^–/–^ and WT mice to examine the impact of CB_1_ receptor gene deletion on the structure and function of the mouse brain. In ANOVA results, genotype, sex, and genotype-sex interaction factors had significant results.

For the genotype factor alone, only 2 brain regions (SS, AUD) had a significant difference in RD between *cnr1*^–/–^ and WT mice (Table 1) (ANOVA genotype factor, SGoF corrected *p* < 0.05). FA, AD, and MD had no significant differences (ANOVA genotype factor not significant).

For the sex factor alone, six brain regions had a significant difference in RD between *cnr1*^–/–^ and WT mice (Table 1) (ANOVA sex factor, SGoF corrected *p* < 0.05). FA, AD, and MD had no significant differences (ANOVA sex factor not significant).

**Figure 3:**
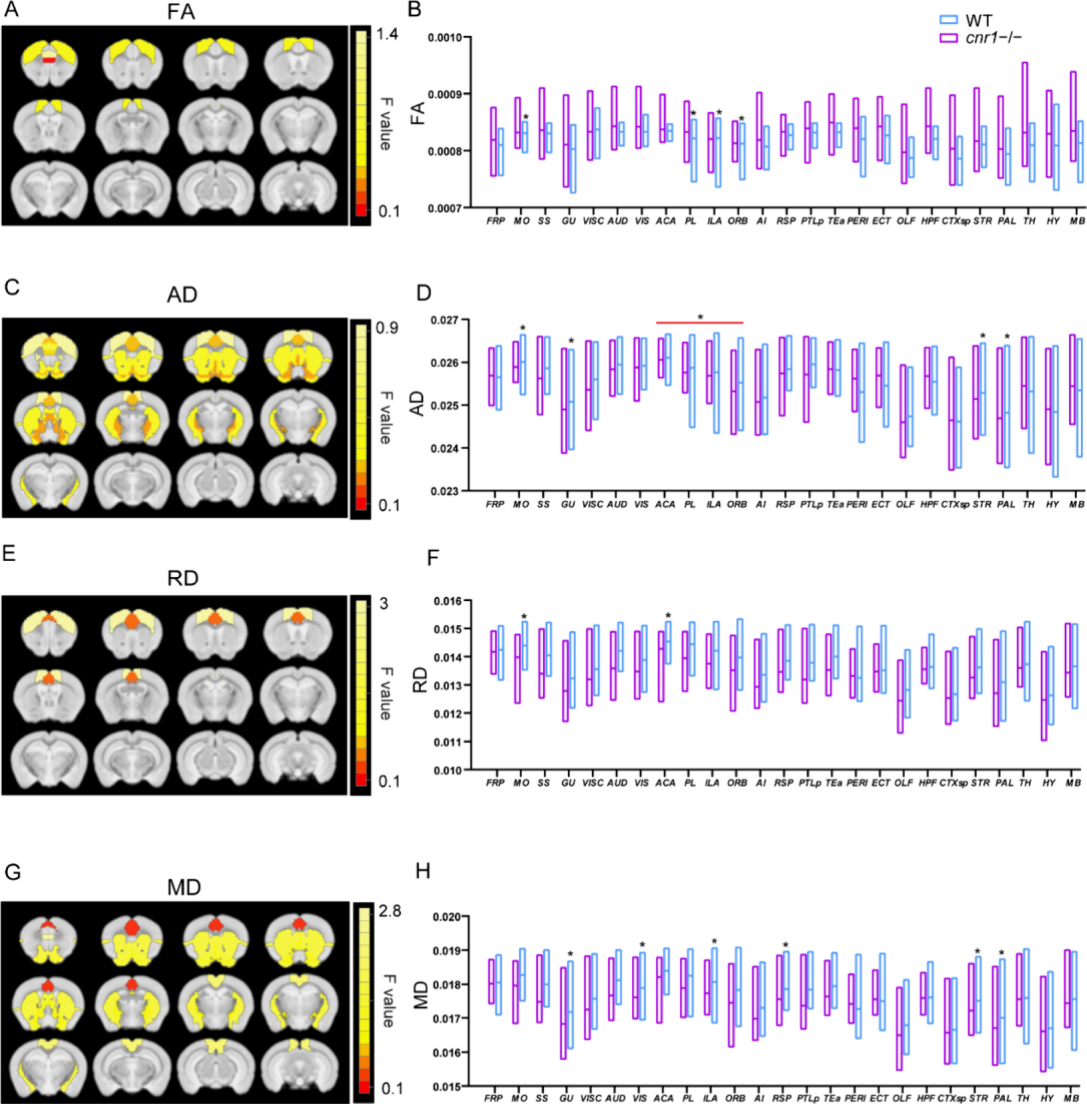
Comparison of spatio-temporal correlation tensor (STCT) parameters between *cnr1*^–/–^ mice and WT mice. Significant brain regions (ANOVA interaction factor, *p* < 0.05, SGoF corrected) have been colored based on that region’s *F* value and superimposed over the T2 template image. Twelve slices are shown from rostral to caudal, top of the brain upward. Results are shown for fractional anisotropy (FA) (A), axial diffusivity (AD) (C), radial diffusivity (RD) (E), and mean diffusivity (MD) (G). Group comparison of FA (B), AD (D), RD (F), and RD (H) in individual brain regions. Data are mean ± s.e.m. (*N* = 12 for *cnr1*^–/–^ and *N* = 12 for WT). * indicates *p* ≤ 0.05 corrected with SGoF. Region of interest names are in Table S1.

For the interaction factor between sex and genotype, many more brain regions were significant across FA, AD, RD, and MD in STCT analysis. For RD, only 2 brain regions (MO, ACA) had significant differences between *cnr1*^–/–^ mice and WT mice under the interaction of genotype and sex factors (Table 1, Figure 3). However, in FA, there were 4 brain regions with significant differences, in AD, there were 8 brain regions, and in MD, there were 7 brain regions, under the interaction of genotype and sex factors (Table 1, Figure 3), despite these metrics not having any brain regions significant under genotype or sex factors alone (Table 1) (ANOVA interaction factor, SGoF corrected *p* < 0.05).

### DTI vs STCT Results

**Figure 4:**
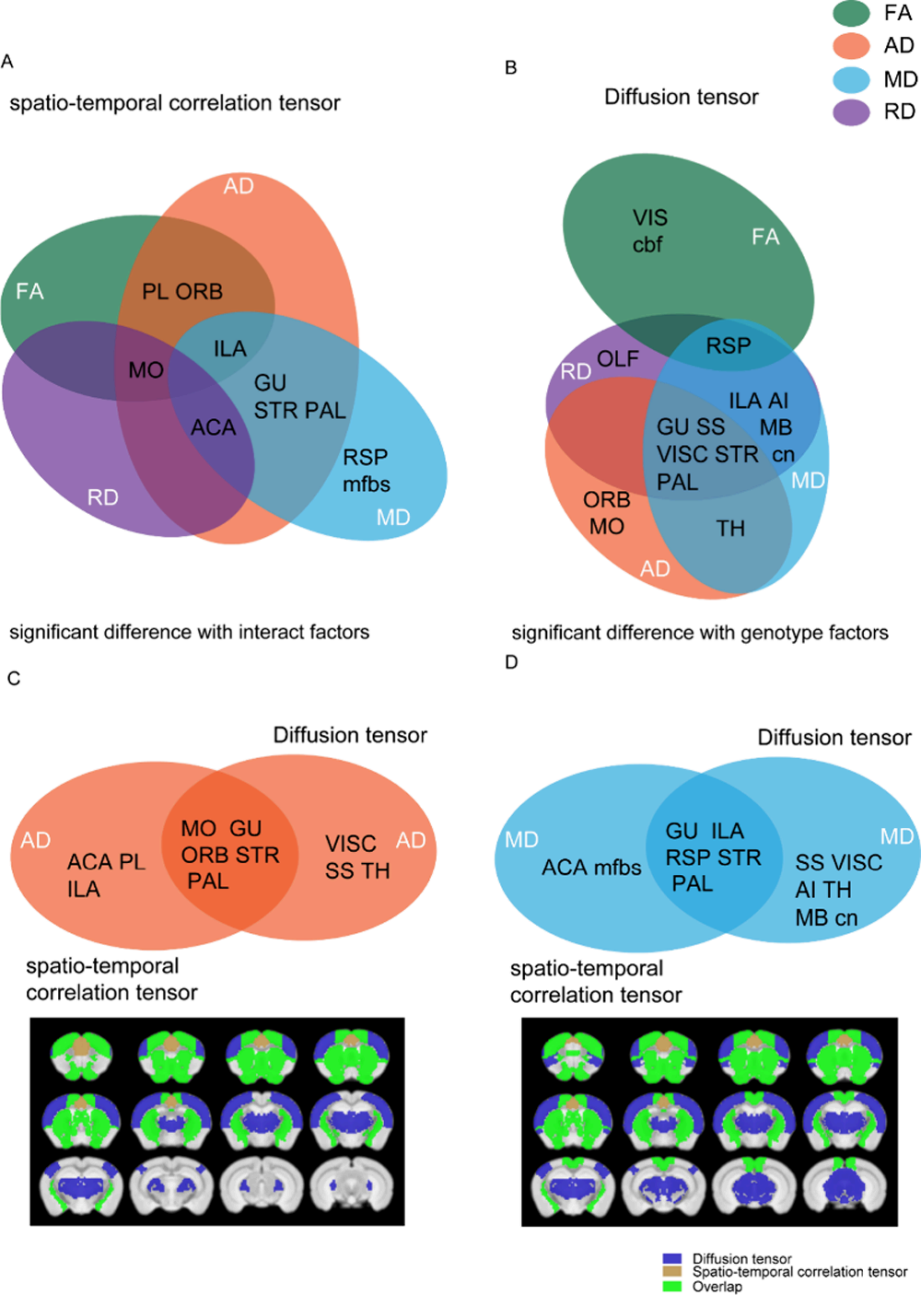
Summary of the significant brain regions for *cnr1*^*–/–*^ mice vs WT mice common between DTI vs STCT. Results from DTI are significant for ANOVA genotype factor, and results from STCT are significant for the interaction factor. Brain regions are shown in black text inside circles, metric is shown in white text inside circles, modality is shown outside circles. (A) STCT modality, four metrics (FA, MD, AD, RD) compared (B) DTI modality, four metrics (FA, MD, AD, RD) compared (C) AD metric, DTI and STCT modalities compared (D) MD metric, and DTI and STCT modalities compared. Significant brain regions in AD (left) and MD (right), have been colored and superimposed over the T2 template image. (Significant white matter regions are not shown here.) Blue is DTI, khaki is STCT, and green is the overlap between DTI and STCT. Abbreviations for brain regions: somatomotor areas (MO), somatomotor areas (SS), gustatory areas (GU), visceral area (VISC), visual areas (VIS), prelimbic area (PL), infralimbic area (ILA), orbital areas (ORB), anterior cingulate area (ACA), agranular insular area (AI), retrosplenial area (RSP), olfactory areas (OLF), striatum (STR), pallidum (PAL), thalamus (TH), midbrain (MB), cranial nerves (cn), cerebellum related fiber tracts (cbf), medial forebrain bundle system (mfbs).

Results from DTI and STCT are shown side by side in Table 1, and Venn diagrams are shown in Figure 4.

The DTI modality with the genotype factor (Figure 4B) shows substantial overlap in terms of significant brain regions for the metrics of MD, RD, and AD. The FA metric shows less overlap, although the RSP brain region overlaps with RD and MD. The STCT modality with interaction factor (Figure 4A) shows overlap for 2–3 metrics for the most significant brain regions, with the most overlap for the MD and AD metrics and the AD and FA metrics. This result indicated substantial overlap within each modality between the 4 metrics for the different brain regions.

We also compared significant brain regions for DTI for the genotype factor to STCT for the interaction factor. Results from comparing AD (Figure 4C) and MD (Figure 4D) had substantial overlap in terms of significant brain regions. This result indicated that, while DTI measures the brain structure and STCT measures the brain function, the change they measured in WT vs *cnr1*^*–/–*^ mice was substantially similar.

### Genotype-Sex Interaction in STCT Results

**Figure 5:**
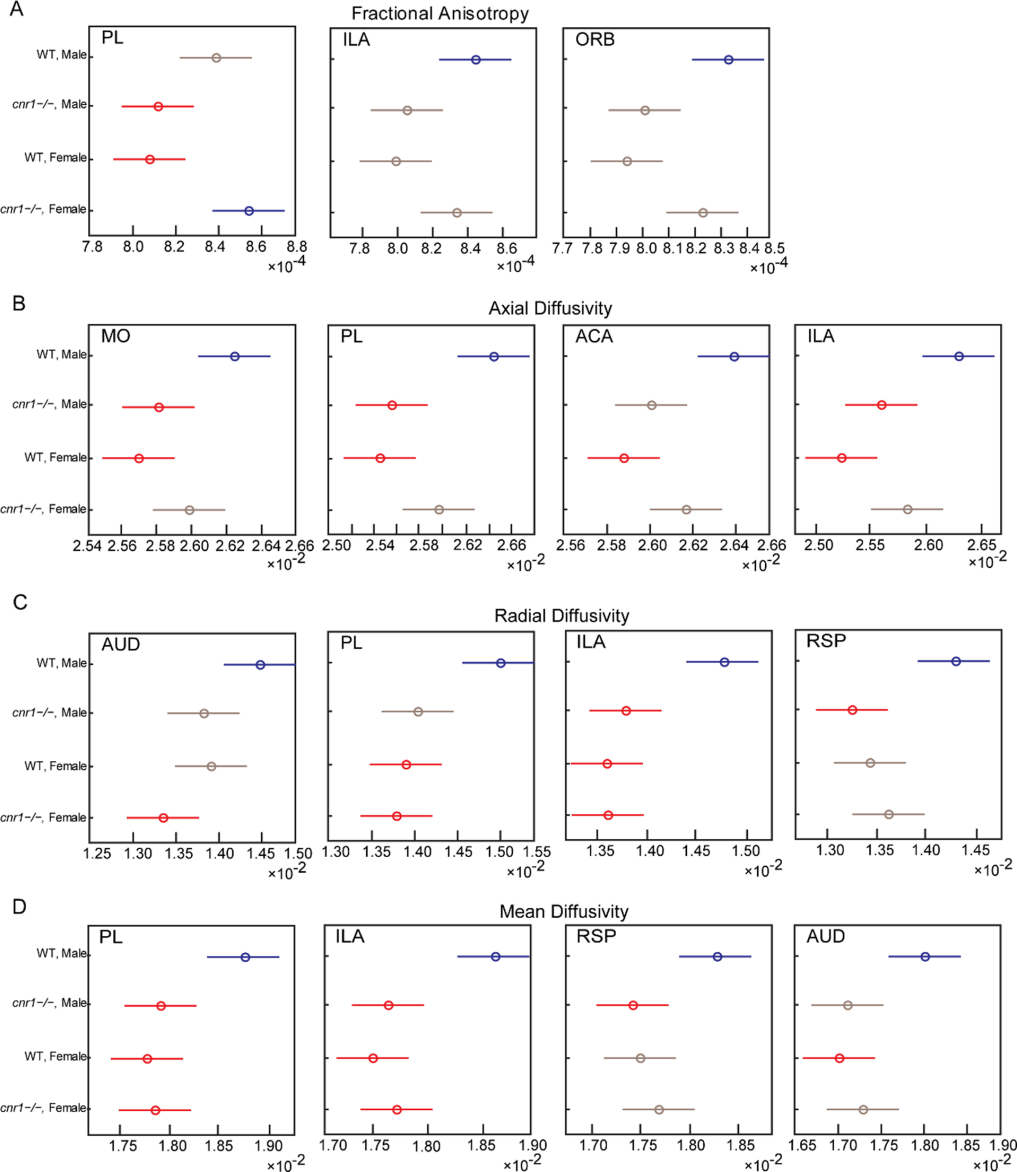
Comparisons of STCT calculated FA (A), AD (B), RD (C), and MD (D) in selected brain regions with STCT analysis. *N* = 24, 6 male WT, 6 female WT, 6 male *cnr1*^–/–^, 6 female *cnr1*^–/–^. The highest mean group is shown in blue. Groups shown in red are significantly different than the blue group (*p* < 0.05, corrected with Tukey’s honestly significant difference procedure). Groups shown in gray were not significantly different from the blue group.

Unlike DTI, where significant results were primarily the ANOVA genotype factor, STCT significant results were primarily the ANOVA interaction factor, genotype vs sex interaction. To understand why this was the case, we selected representative brain regions that had statistically significant differences in STCT parameters between different groups (Figure 5). We found that WT males were significantly higher than all other groups with the exception of the FA in PL of female *cnr1*^–/–^ mice, which was significantly greater than that of male *cnr1*^–/–^ mice and female WT mice. This indicated that STCT in male mice was more affected by the *cnr1* gene deletion than in female mice and that the effect of the gender factor was stronger within the same genotype, primarily in WT mice.

### Pharmacological MRI (phMRI) of Rimonabant

To compare the long-term elimination of CB_1_ to short-term inhibition of CB_1_ we used rimonabant, an inverse agonist of CB_1_ with high binding affinity, to map responsive brain regions using phMRI in WT mice and compare to prior results.

**Figure 6:**
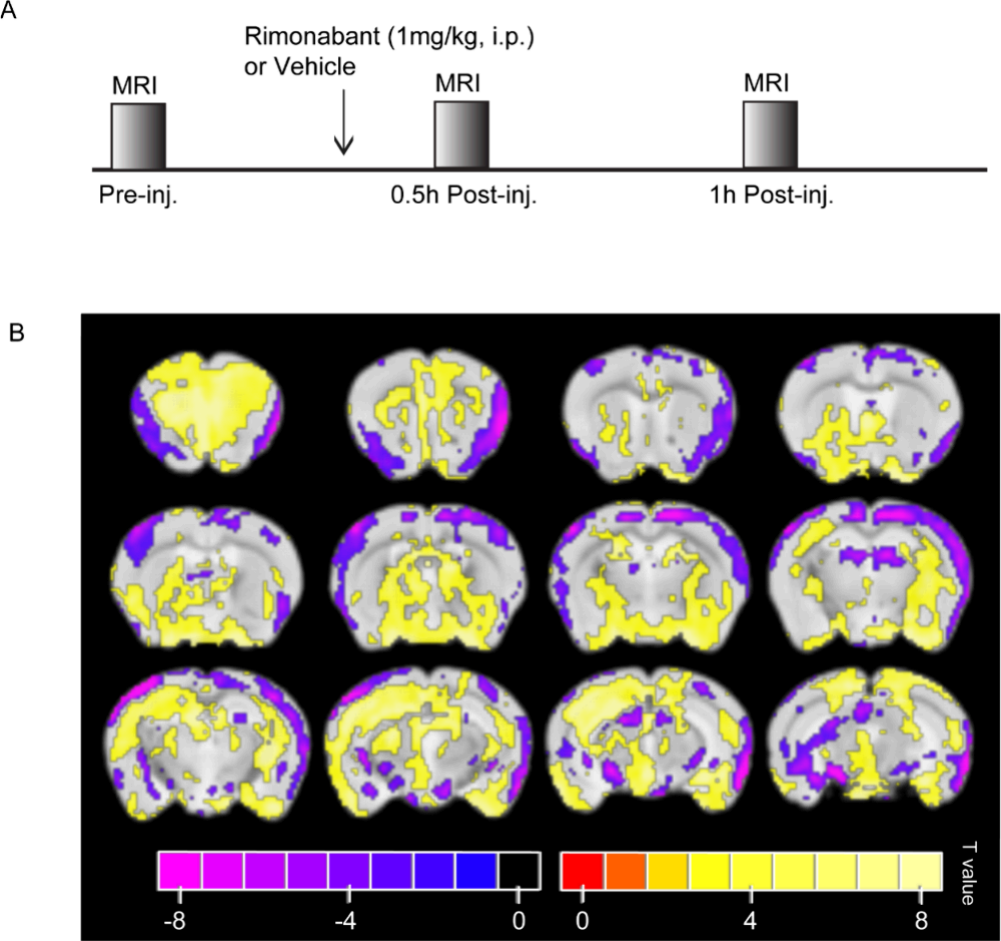
Rimonabant administration induced BOLD positive and negative activation in the brain. (A) Scheme of the mouse drug challenge and MR scanning timeline. (B) Group (*n* = 8/group) statistical parametric maps showing changes in BOLD contrast with a significance threshold set to *p* < 0.05 (corrected with SGoF) following acute administration of rimonabant (1 mg/kg body weight, i.p.), baseline vs 60 min (first level), vehicle vs rimonabant (second level). Blobs in warm colors (right) indicate regions of increased BOLD signal compared with vehicle, whereas blobs in cool colors (left) are regions of decreased BOLD signal.

Using a recent high-sensitivity method of generating statistical brain activation maps,^36^ we identified the parts of the brain where an effect had occurred a certain amount of time after administering the vehicle or rimonabant (first level) and where there was a difference between the drug and vehicle’s first level results (second level). The scheme of mouse drug challenge and MR scanning timeline are shown in Figure 6A. We collected EPI images immediately prior to the drug injection as well as at 30 and 60 min after the injection. As results may not have been stable at 30 min,^37^ we focused on results at 60 min. The second level map of statistically significant *T* values 60 min after the rimonabant or vehicle injection is shown in Figure 6B.

At 60 min after injection, many regions with both significant positive and significant negative activations can be observed. Regions that included at least 100 voxels of positive activation were the secondary motor areas (layers 2, 3, and 5), the Hippocampus (subregions CA1 and CA3), the molecular layer of the dentate gyrus, the caudoputamen, the medial amygdalar nucleus, and the olfactory tubercle and main olfactory bulb. Regions that included at least 100 voxels of negative activation were the primary somatosensory areas (layers 2–3), the midbrain reticular nucleus, and periaqueductal gray matter. Regions that included both at least 100 voxels of positive and 100 voxels of negative activation were the piriform area and the midbrain. (Possibly unstable) Results 30 min after injection showed positive activation across almost the entire brain, with the exception of entorhinal areas, which had negative activation (Figure S4).

### rs-fMRI and STCT Under Rimonabant

**Figure 7:**
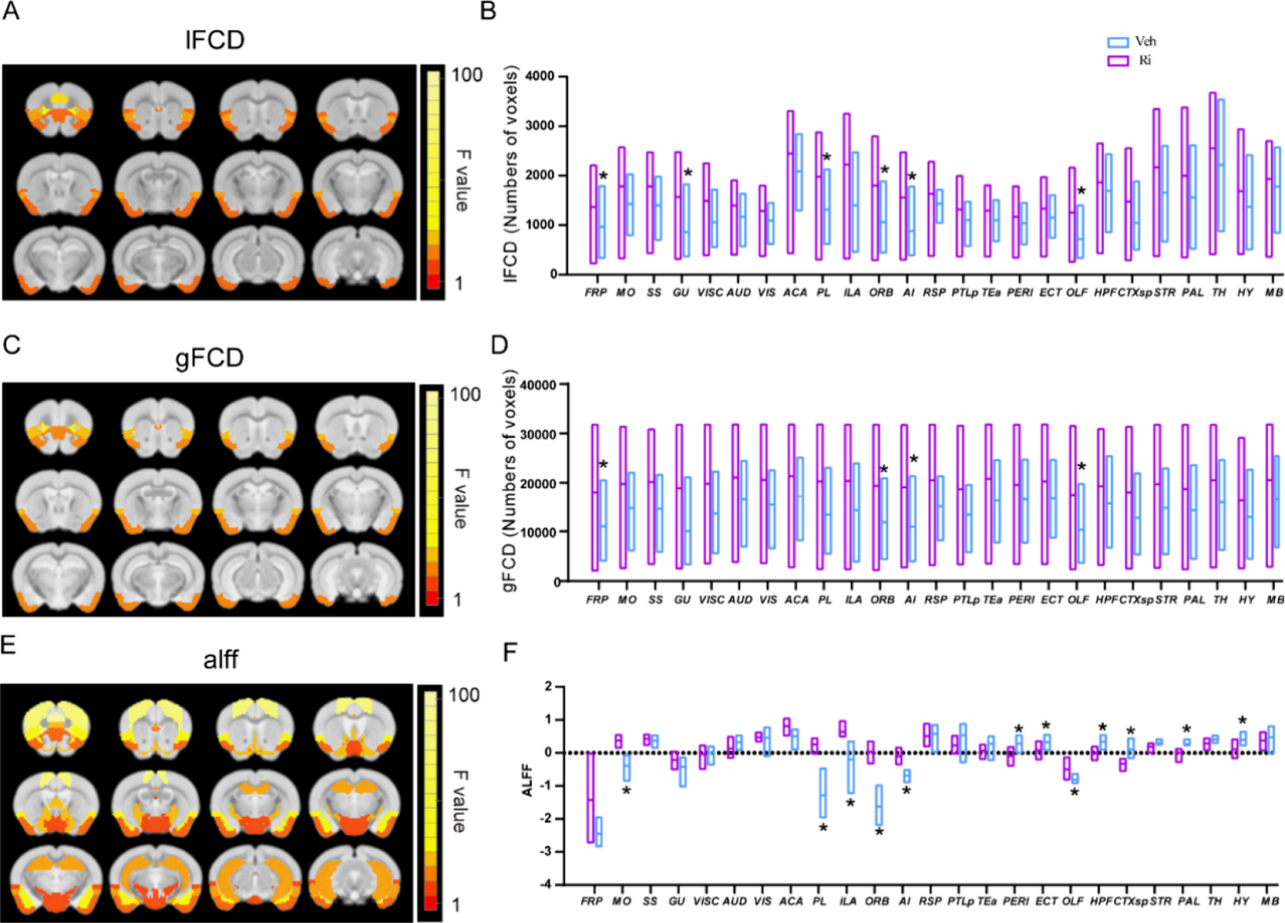
Comparison of functional connectivity density (FCD) and amplitude of low-frequency fluctuations (ALFF) between Rimonabant and vehicle administration in mice at 60 min. Significant brain regions (ANOVA drug factor, *p* < 0.05, SGoF corrected) have been colored based on that region’s *F* value and superimposed over the T2 template image. Twelve slices are shown from rostral to caudal, top of the brain upward. Results are shown for local FCD (lFCD) (A), global FCD (gFCD) (C), and ALFF (E). Group comparison of lFCD (B), gFCD (D), and ALFF (F) in individual brain regions. Data are mean ± s.e.m. (*N* = 8 for the Rimonabant group and *N* = 8 for the vehicle group). *Indicates *p* ≤ 0.05 corrected with SGoF. Region of interest names are in Table S1.

We also examined the difference in network activity between rimonabant and vehicle at 60 min by comparing the fMRI time courses directly to rs-fMRI data (rather than in SPM12). First, we calculated resting-state functional connectivity correlation coefficients. There was no statistically significant difference between rimonabant and the vehicle group in correlation coefficients within the 25 gray matter regions (Figure S5). Next, we compared averages in each brain region, calculated from per-voxel maps for ALFF, gFCD, and lFCD. As compared to the vehicle group, the results indicated that rimonabant administration may increase FCD values at 60 min, both gFCD and lFCD. The following brain regions had a significant difference between rimonabant and vehicle: FRP, ORB, AI, and OLF in both gFCD and lFCD, and GU, PL only in lFCD (ANOVA drug factor, SGoF corrected *p* < 0.05, sex and interaction factors not significant) (Figure 7A–D). Under rimonabant, ALFF was lower than under vehicle in mostly subcortical regions, including HFP, OLF, CTXsp, STR, and HY, and higher than under vehicle in mostly cortical regions, including MO, PL, ILA, ORB, and AI (ANOVA drug factor, SGoF corrected *p* < 0.05, sex and interaction factors not significant) (Figure 7E, F).

We also checked the functional directional uniformity with STCT. Using STCT, FA, AD, RD, and MD were marginally higher in the rimonabant group but were not statistically significantly different from the vehicle group (ANOVA drug, sex, and interaction factors not significant) (Figure S6).

### Comparison between Modalities and Time Scales

**Figure 8:**
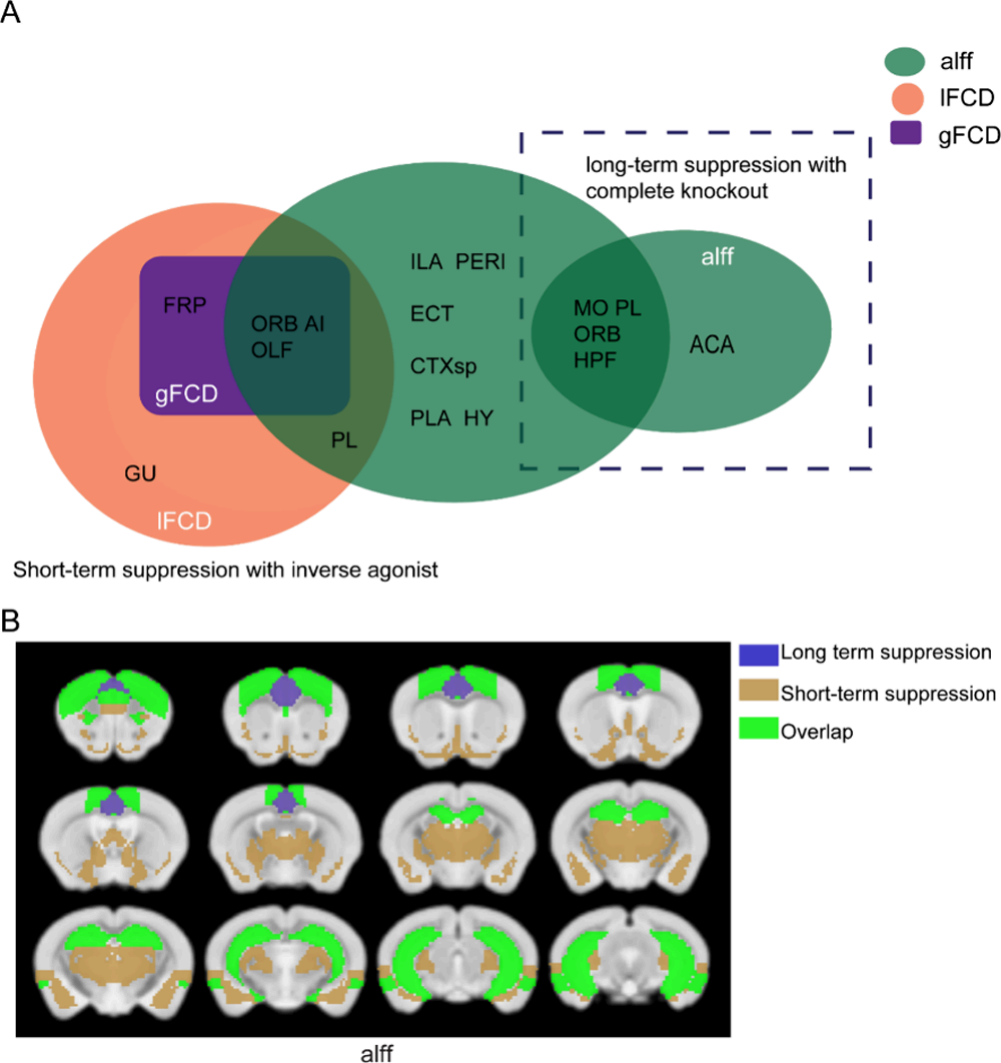
Summary of the significant brain regions common between short-term suppression with an inverse agonist (rimonabant) and long-term suppression with complete knockout (*cnr1*^*–/–*^). Results from *cnr1*^*–/–*^ vs WT are significant for the ANOVA genotype factor, results from rimonabant vs vehicle are significant for the ANOVA drug factor. (A) Brain regions are shown in black text inside circles, the metric is shown in white text inside circles, and the modality is shown outside circles. ALFF, gFCD, and lFCD from rimonabant vs vehicle are compared to ALFF from *cnr1*^*–/–*^ vs WT. (B) Significant brain regions in ALFF have been colored and superimposed over the T2 template image. Blue is *cnr*^*–/–*^ versus WT (long-term suppression), khaki is rimonabant versus vehicle (short-term suppression), and green is an overlap between the two comparisons. Abbreviations for brain regions: frontal pole (FRP), somatomotor areas (MO), Gustatory areas (GU), prelimbic area (PL), infralimbic area (ILA), orbital areas (ORB), perirhinal area (PEPI), ectorhinal area (ECT), anterior cingulate area (ACA), agranular insular area (AI), hippocampal formation (HPF), olfactory areas (OLF), cortical subplate (CTXsp), pallidum (PAL), hypothalamus (HY).

A Venn diagram is shown in Figure 8 to compare rs-fMRI results from rimonabant vs vehicle at 60 min to *cnr1*^*–/–*^ vs WT in terms of significant brain regions. Whereas rs-fMRI showed few significant differences in *cnr1*^–/–^ mice, and only in the ALFF metric, mice under rimonabant had many more significant differences under ALFF and both types of FCD. Only two brain regions overlapped between short-term (rimonabant) and long-term (*cnr1* knockout) suppression, HPF and MO. Notably, these are both regions of very high CB1 concentration, being located in the hippocampus and cerebral cortex, respectively (Figure S7).

## Discussion

### Behavioral Relevance of Brain Regions Altered in *cnr1*^*–/–*^

When comparing adult *cnr1*^*–/–*^ mice to WT mice, we saw no significant differences in standard functional connectivity, no differences in FCD, and only a few regions with altered ALFF. However, considering DTI and STCT, many brain regions showed altered FA, MD, AD or RD. There was substantial overlap between significant brain regions between these four metrics (Figure 4A,B) and also within each metric, there was a large overlap between the DTI and STCT modalities (Figure 4C,D). Our results thus suggest that brain network function itself may not be substantially different in *cnr1*^*–/–*^ mice, but the physical structure of white matter along which the networks communicate (measured with DTI) as well as the active direction of communication within those networks (measured with STCT) is altered.

*cnr1*^*–/–*^ mice are generally as healthy as WT mice, including normal blood pressure and heart rate,^38^ and normal pain response under some tests^38,39^ (although not some other tests^40^). However, previous studies have found subtle variations in behavior^17^ which we can compare to our results. Indeed, many of the brain regions where we found significance using DTI and STCT have been previously linked to altered behavior in the *cnr1*^*–/–*^ genotype.

The retrosplenial cortex (RSP) is believed to be focused on translating between egocentric, internal, information and allocentric, external, information, e.g., using a mental map to navigate space in reality.^41−44^ Dense connections to the visual cortex (VIS)^45^ indicate the role of vision in building these internal models of the external world. A previous study demonstrated altered visual field activation in *cnr1*^–/–^ mice,^15^ and behavioral studies where *cnr1*^–/–^ mice have improved performance in object recognition tasks where they can use visual information.^46,47^

The infralimbic cortex (ILA) is primarily indicated in emotional regulation^48^ and extinction of behavior.^49^ “Extinction” here refers to the gradual loss of a conditioned response to a stimulus when it is no longer reinforced. Several studies have shown deficits in extinction in *cnr1*^–/–^ mice^16,29^ and modulation of extinction by THC.^50,51^

The brain regions implicated were not all high in CB_1_ density, for example, the thalamus (TH) (Figure S7). However, CB_1_ is still present in the thalamus at low levels, and is relevant in e.g., pain control.^30^

### Structure vs Function in Sex Differences in the *cnr1*^*–/–*^ Genotype

Functional and structural connectivity is known to disassociate in psychiatric diseases, in particular, in schizophrenia. In schizophrenia, some studies have shown no significant correlation between DTI and fMRI measures.^52,53^ The structural abnormalities in schizophrenia have been hypothesized to be due to altered development attempting to preserve normal brain function as best as possible in the disrupted neural state.^54^ Notably, schizophrenia is also related to many genes related to the cerebrum (e.g., ref (55)). Therefore, given some of the results of our study, where structural and functional connectivity diverge, this may be a reasonable hypothesis for chronic CB1 loss also. However, future work is needed to determine this.

Regarding *cnr1*^*–/–*^ mice, they are generally as healthy as WT mice, including normal blood pressure and heart rate, and show only subtle variations in behavior.^56^ During the developmental periods, *cnr1*^*–/–*^ mice might be regulated by neuroplasticity and negative feedback to maintain normal physiological function, whereas, regarding brain structure, *cnr1*^*–/–*^ mice may show microstructural abnormalities from a young age.

While the brain regions found to be significant by DTI and STCT for *cnr1*^*–/–*^ mice vs WT mice had substantial overlap, the major difference between the two modalities was that DTI found brain regions significant for the genotype factor and STCT found brain regions significant for interaction between genotype and sex. Further investigation revealed that this was because male *cnr1*^*–/–*^ mice were different from male WT mice, but female mice of the two genotypes differed far less (Figure 5). Our results thus suggest that while the physical structure was altered in both sexes, the activity upon that structure was altered more in male mice.

Previous research suggests that the distribution of CB_1_ receptor expression and function differs by sex-related variation in healthy, wild-type individuals across species.^57,58^ Hungund et al. found, similar to our results, that *cnr1* knockout eliminated a sex-based difference, in their study it was a behavior, however, wherein WT female mice consumed more alcohol than WT males but it was more equal in *cnr1*^*–/–*^ males and females.^59^ Coleman et al. found that male mice were more affected than female mice by adolescent cannabis exposure, including FA in the forebrain and hindbrain.^60^ Our results support these studies by suggesting that while male and female mice are both affected by long-term cannabinoid changes in the brain, the effect is greater in males and is observable in adult brain function rather than only as structural changes supporting similar functions.

### Rimonabant Activates the Brain and Alters Functional Connectivity

In mice at 30 min post rimonabant exposure, there was significant positive activation over most of the brain except for entorhinal areas, which showed significant negative activation (Figure S4). However, at 60 min post rimonabant exposure, there was a range of both significant positive and significant negative activation. Based on work by Rinaldi-Carmona et al. results may not have been stable at 30 min,^37^ thus in the main text we focused on 60 min.

At 60 min, regions of positive activation tended to be regions with higher CB_1_ concentration than regions of negative activation (Figure S7), similar to rs-fMRI results. The somatosensory cortex and entorhinal cortices were exceptions. Potentially the somatosensory cortex may be due to somatosensory-motor disconnection under anesthesia. The entorhinal areas showed significant negative activation at both time points, possibly due to CB_1_ in these regions having a key role in modulating network oscillatory activity.^61^ Significant results in rats found by Dodd et al. at 12–22 min post-exposure were similar to our results in mice for at 60 min post-exposure, the dose was 1 mg/kg in both species.^62^ However, in mice, the results were not limited to a relatively small and ventral portion of the brain as they were in rats. This may be due to higher sensitivity in our study due to the higher field MRI (9.4 T vs 7 T) and the high sensitivity method used by Sanganahalli et al.^36^

Regarding functional connectivity, as a strong inverse agonist, rimonabant’s effects might be predicted as affecting similar brain regions as a strong agonist such as THC. THC tends to increase functional brain connectivity, focused in brain regions with high densities of CB_1_-receptors.^28^ Our observation of network changes in high CB_1_ density regions supports this. Indeed, the brain regions we observed as significant in short-term suppression using FCD or ALFF in mice overlap with the networks observed altered under human exposure to THC (FRP, PL, ILA, ORB, PERI, ECT, HPF), and regions significant under rimonabant in mice but not under THC in humans tend to be small and difficult to image in humans (GU, OLF), or do not exist in adult humans (CTXsp).^28^ Notably, Klumpers et al. hypothesized but did not see the hypothalamus (HY) being affected but it was affected by rimonabant in our study.

### Major Difference between Long-Term and Short-Term Suppression

Our initial hypothesis in this work was that the drug and genetic knockout effects would be similar, as stated in our introduction, based on previous use of such mice in studies.^17,39^ However, we discovered this to not be the case except for a few high CB1 concentration brain regions, such as the hippocampus.

As shown in Figure 8, only one fMRI-derived metric (ALFF) for only two brain regions (HPF, MO) overlapped at the standard significance threshold used in our work. However, many more brain regions did not overlap (4 for *cnr1*^*–/–*^ vs WT, 12 for rimonabant vs vehicle), and while short-term suppression showed significant gFCD and lFCD changes, long-term suppression showed no changes to FCD. Notably, ALFF is a highly sensitive metric,^25,32^ and the larger locations of HPF and MO (hippocampus and neocortex) have high CB_1_ density (Figure S7). Thus, other than areas and metrics of high sensitivity, short-term and long-term suppression of cannabinoid signaling affected a very different network of brain regions.

As discussed above, the affected brain network in *cnr1*^*–/–*^ is linked to known behavioral abnormalities in these mice^15,16,29,30^ and related to microscopic changes in white matter.^21^ While a change in one direction in DTI-derived measures is often considered a change in quality, e.g., of myelin integrity or myelin injury^23,24^ we observed measures differing in both directions from WT to *cnr1*^*–/–*^ mice (e.g., RD and MD in Figure 1). It seems likely that, rather than considering the changes in the knockout as pure deficits, it likely represents a reorganization to maintain brain function so that it is not disrupted as seen under rimonabant. Previous studies of FA have found reorganization in the presence of a detrimental alteration.^55,66^ This can thus provide a hypothesis for *cnr1* knockout when moving forward from the present study.

### Limitations

While we were careful to use identical imaging protocols and anesthesia for all groups, using a surface coil was necessary to achieve the SNR needed by our study. Therefore, different parts of the brain would have different SNRs. Because of this, only statistical comparisons between different groups were performed, and we did not perform statistical comparisons between different brain regions.

Compared to DTI-derived tensors or the ALFF and FCD metrics in fMRI, STCT-based methods have not been heavily studied. When establishing our methods, we observed that excess smoothing on STCT would eliminate all of the significance from these results. Thus, we did not optimize the smoothing parameter but rather kept our methods based on previous mouse work.^67−69^ The effect of smoothing on STCT is an open question that future work can address.

We used urethane anesthetic in our study as it was effective in previous studies that measured brain activity with rs-fMRI in the mouse^54,70,71^ and has little effect on normal cardiovascular and respiratory systems and maintenance of spinal reflexes.^67,72,73^ However, any general anesthetic will alter brain activity. While acceptable for rs-fMRI, urethane may be less effective for translation to electrophysiology as it can show a steady and periodic alternation between slow and fast-wave states.^74^ Due to the toxicity of urethane anesthetic and the stress of imaging upon the physiological condition of the mice, longer time points than 60 min have not been possible with our protocol thus far. Awake imaging could allow longer imaging sessions; however, it requires much more difficult and time-consuming preparation for the mice, and their awake or asleep state will vary.^75^

In rodent research, behavior has differed between acute and chronic rimonabant conditions. Acute dosing with rimonabant produced anorexia and other behavioral disruptions, such as increased scratching, grooming, and wet-dog shakes. Chronic rimonabant produced both anxiogenic and anxiolytic effects.^63^ The endogenous cannabinoid system plays a crucial role in the functional foundations of learning and memory. Acute and chronic blockade of CB1 receptors with rimonabant also improved learning and memory.^76−78^ Continuous pharmacological blockade of the CB1 receptor with rimonabant increased the transcription of genes required for the survival of existing neurons (BDNF), the expression of late-phase LTP (Gria1), and the regulation of axogenesis and synaptogenesis (Syn1), but did not alter the expression of *Cnr1* or *Cnrip1*.^78^ These results suggest that a continuous pharmacological blockade of the CB1 receptor enhanced the expression of genes associated with synaptic plasticity. As our study already used multiple experimental groups, multiple imaging modalities, and multiple metrics calculated from each modality, we focused only on adult mice. However, based on our results, future work studying mouse development (particularly adolescent development considering previous work^56^) is strongly indicated.

## Conclusions

Moving forward, our results provide a concrete network of brain regions for future study. Furthermore, the difference between short-term and long-term suppression suggests that the study of brain development within these brain regions will be necessary to fully understand cannabinoid signaling across the whole brain.

Contrary to our hypothesis, we observed substantial differences between the “short-term” administration of rimonabant versus “long-term” complete *cnr1* knockout. Instead, our results suggest that the chronic loss of *cnr1* is substantially different from short-term suppression, subtly rewiring the brain but largely maintaining network activity.

In human studies, there is much interest in both pathological mutations in CB_1_ (e.g., chronic pain^79^) and clinical use of CB_1_ suppression.^18,19^ However, our results indicate that short-term suppression of cannabinoid signaling does not necessarily give insight into the brain networks involved in long-term suppression of cannabinoid signaling and vice versa. Furthermore, our results also further underscore that researchers of cannabinoid signaling need to be aware of sex-specific effects regardless of the animal model they study. For these reasons, we thus urge more preclinical studies for any drugs that suppress CB_1_.

## Materials and Methods

### Ethical Approval

All experimental procedures were approved by the Institutional Animal Care and Use Committee of ShanghaiTech University, China, and Fudan University, China, in accordance with the National Institutes of Health guide for the care and use of Laboratory animals.^80^ Animal data reporting herein follows the ARRIVE 2.0 guidelines.^81^

### Generation of *cnr1*^*–/–*^ Mice

*cnr1*^*–/–*^ mice were generated as described before.^21^ Briefly, for *cnr1*^*–/–*^ 1 gene targeting, two sgRNAs were designed to target either the upstream or downstream region of its coding sequence by the CRISPR design tool (http://crispr.mit.edu) and screened for on-target activity using UCATM (Universal CRISPR Activity Assay, Biocytogen). PCR amplification was performed to obtain the T7-Cas/sgRNA PCR products. They were used as the template for in vitro transcription and obtained the Cas9 mRNA and sgRNA products. C57BL/6 female mice and KM mouse strains were used as embryo donors and pseudopregnant foster mothers, respectively. Cas9 mRNA and sgRNAs were injected into the cytoplasm of one-cell-stage fertilized eggs. After injection, surviving zygotes were transferred into oviducts of KM albino pseudopregnant females and allowed to develop to term. Mutant mice were genotyped to ensure the deletion of the target CB1 segment.

### Animals

Male and female C57BL/6J mice (wild-type, WT) and male and female *cnr1*^–/–^ mice (*cnr1*^–/–^, Biocytogen, based on C57BL/6J) of 8–12 weeks of age weighing 20–30g were included in this study. Animals were housed under a 12 h light/dark cycle at a room temperature of 22 ± 1 °C with 45% humidity and given ad libitum access to food and water.

The total number of mice imaged was 40. Twelve WT mice (6 male) and 12 *cnr1*^*–/–*^ mice (6 male) were imaged without pharmacological intervention. Sixteen WT mice (8 male) were imaged under the pharmacological MRI (phMRI) protocol, of these 8 received rimonabant (4 male), and the remainder received vehicle.

### MRI Data Acquisition

Mice were imaged with MRI. The MRI data were acquired using a Bruker BioSpec 11.7T scanner (Bruker, Ettingen, Germany) equipped with an 89 mm volume coil for transmission and a 4-channel phased-array *CryoProbe* (Bruker, Ettingen, Germany) mouse head coil for receiving. Animals were anesthetized with 25% urethane dissolved in distilled water (Sigma, U2500–100G) using intraperitoneal bolus injections (1.3 g/kg) divided into three separate doses. The interval time was 10 min between each dose.^54,67,70^ No ventilation is needed to maintain the hemodynamics under urethane. Sufficient anesthesia was judged by a no-toe-pinch reflex or weak-toe-pinch response. A built-in warm water heating pad (Bruker) was used to maintain body temperature between 36 and 37.5 °C. Respiratory rates were monitored throughout, and respiration was maintained within the range of 180–220 breaths per minute.

After a localizer scan for animal positioning, a T2-weighted MRI (T2WI) rapid acquisition with relaxation enhancement (RARE) scan was acquired with the following parameters: echo time (TE) = 30 ms, repetition time (TR) = 4600 ms, field of view (FOV) = 18 × 18 mm^2^, matrix size = 256 × 256 (∼70 μm in-plane resolution), slice thickness = 0.3 mm, No. of slices = 48, average = 2, RARE factor = 8. For characterizing the microstructure of the axon, diffusion tensor imaging (DTI) was acquired with spin–echo echo-planar imaging (EPI) with the following parameters: FOV = 18 × 18 mm^2^, matrix = 128 × 64, No. of slices = 36, slice thickness = 0.30 mm with 0.10 mm gap, voxel size = 0.14 × 0.28 × 0.30 mm^3^, TE/TR = 24/2500 ms, *b* = 1000 s/mm^2^, 30 diffusion directions, 5 b = 0 s/mm^2^ images, No. of segments = 4, averages = 6, total scan time = 35 min. For measurement of resting state functional connectivity, resting-state functional MRI (rs-fMRI) blood-oxygen level-dependent (BOLD) data were acquired using gradient-echo EPI with the following parameters: TE/TR = 11.3/1000 ms, FOV = 18 × 18 mm^2^, matrix size = 90 × 90 (∼89 μm in-plane resolution), slice thickness = 0.5 mm, No. of slices = 16, number of repetitions = 480, total scan time = 8 min.

### Data-Driven MRI Analysis

#### Diffusion Tensor Imaging (DTI)

The DTI data set was preprocessed in the DSI studio (Version 2022 Jul).^82^ Motion and Eddy current corrections were performed. The DTI data set was exported in the format of 4D NIFTI images and reconstructed from 128 × 64 × 36 to 120 × 120 × 72, then voxel size = 0.15 × 0.15 × 0.15 mm^3^. Summed DWI was exported and used for brain extraction in BrainSuite (Version 21a) (https://brainsuite.org/). Then the DTI data set was denoised using nlsam.

For the analysis of DTI metrics, fractional anisotropy (FA, indicator of the axon integrity), mean diffusivity (MD), axial diffusivity (AD, indicator of axon injury), and radial diffusivity (RD, indicator of myelination) were calculated in the DSI studio (Version 2022 Jul)^82^ using the denoised DTI data set. QSDR reconstruction was adapted using an ABA mouse brain template. Then, the mean parametric values of each 3D brain region were extracted in Matlab (Mathworks, R2021b).

#### Resting State Functional MRI (rs-fMRI)

rs-fMRI BOLD data were preprocessed and analyzed using custom-written scripts in MATLAB. Motion correction and slice timing were performed in SPM12 (Wellcome Trust Centre for Neuroimaging, London, U.K.). Anatomical images were registered to the same mouse brain template as DTI processing used using a nonlinear registration (50 iterations, normalized mutual information, and otherwise default) with BioImage Suite (Yale School of Medicine, 2015; bioimagesuite.yale.edu). Each mouse’s corresponding nonlinear registration was then applied to rs-fMRI data from the same mouse. To facilitate intermice statistics, each rs-fMRI image was spatially smoothed (0.5 mm isotropic Gaussian Kernel) and each voxel’s time series was bandpass filtered from 0.01 to 0.3 Hz.^67−69^

In order to reduce noise and improve the quality of the BOLD signal, group-independent component analysis (ICA) was performed in EPI images using the GIFT toolbox (http://mialab.mrn.org/software/gift/).^83^ The number of independent components estimated by the MDL criteria is 5. Among them, components 4 and 5 were removed because they were recognized as the signals from ventricles and white matter (The full components, kept and removed, are shown in Figure S1).

To further increase BOLD signal quality, epochs of the BOLD signal with a low signal-to-noise ratio (SNR) were removed. Because the primary somatosensory cortex of the forelimb (S1FL) has a high correlation between the left and right hemispheres,^84^ lacking correlation in S1FL is a sign of a lack of brain networks in general, indicating a potential problem in the physiological state. rs-fMRI time courses from S1FL were thus divided into five epochs of equal length and correlated to each other. Epochs, where the Pearson’s correlation coefficient (PCC) between bilateral S1FL was lower than 0.3, were removed prior to functional connectivity analysis as was done in previous work.^54,70^

From rs-fMRI data, correlation coefficients were calculated between the average time courses of 25 gray matter regions of interest (ROIs) (see Table S1). Two types of functional connectivity calculations were performed: (1) Calculate the correlation between different brain regions based on unilateral data such as left RSP and left ECT. (2) Calculate the correlation in the same brain region based on bilateral data, such as left RSP and right RSP. A normalizing version of Fisher’s transformation was applied to normalize the Pearson correlation to a hypothetical *N*(0,1) distribution of *Z* scores.^85^ The normality of correlation coefficients within the *cnr1*^–/–^ group and the WT group were each tested, and then One-way ANOVA with interaction between groups was performed. The *p*-value threshold of 0.05 was corrected for Type 1 errors based on the SGoF FWER test.^86^ To calculate correlation coefficients within groups, the normalized correlation coefficient matrix of *Z* values was averaged across all mice, and then, the inverse of the normalizing Fisher transform was used to obtain the average *r*-score matrix.

#### Per-Voxel rs-fMRI Metrics

The amplitude of low-frequency fluctuations (ALFF) in functional magnetic resonance imaging (fMRI) is a widely used metric for assessing spontaneous neuronal activity.^87^ ALFF provides insights into intrinsic brain activity and has been widely used in studies investigating cognitive processes, neurological disorders, and psychiatric conditions.^88,89^ For each voxel, the power spectrum is computed from the fMRI time series, and then the square root of the power spectrum is obtained. The ALFF value is derived by averaging the square root values within the frequency range of 0.01–0.1 Hz.^32^ Results are averaged together for all voxels within a specific brain region.

Functional connectivity density (FCD) mapping, a metric derived from graph theory and employing rs-fMRI data, utilizes Pearson’s correlation to generate a map of functional connections in the brain. It quantifies the number of local and global functional connections for each voxel. Local functional connectivity density (lFCD) assesses connections within a nearby cluster of voxels, while global functional connectivity density (gFCD) measures connections across the entire brain. In this study, we employed lFCD-based FCD and utilized a correlation threshold of *r* ≥ 0.25 to determine the number of voxels linked to a specific voxel. The nearby cluster size was set at a radius of 2 mm.^90^ Similar to lFCD, gFCD also used a correlation threshold of *r* ≥ 0.25 to determine the number of voxels linked to the entire brain. The resulting lFCD and gFCD values were calculated on a per-mouse basis. Results are averaged together for all voxels within a specific brain region.

#### Spatio-temporal Correlation Tensor

As per Ding et al.,^34^ using rs-fMRI data, a set of unit vectors for each voxel that points to the 26 nearest voxels in the local 3 × 3 × 3 neighborhood is defined, and the temporal correlation of BOLD signals along each of the directions is computed. The spatio-temporal correlation tensor (STCT), *T*, is computed by solving:
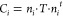
where *C_i_* is the correlation coefficient along the direction *n_i_*.

*T* characterizes the local profile of temporal correlations in MRI signals with the major eigenvector representing the dominant direction of temporal correlations. In this experiment, we computed the eigenvectors ε1, ε2, and ε3 and the eigenvalues λ1, λ2, and λ3 of the tensor *T*. Similar to the diffusion tensor used in DTI analysis above, FA, AD, RD, and MD values were calculated along with their mean values in different brain regions. STCT data were analyzed in Matlab.

#### Pharmacological MRI (phMRI)

According to the previous studies, WT mice in the phMRI group received rimonabant (1 mg/kg) or vehicle with intraperitoneal injection. Functional EPI images of mice were collected before drug administration, 30 min after drug administration, and 60 min after drug administration.^37,62,91^ The scan parameters are as followed: TE/TR = 11.3/1000 ms, FOV = 18 × 18 mm^2^, matrix size = 90 × 90 (∼89 μm in-plane resolution), slice thickness = 0.5 mm, No. of slices = 16, number of repetitions = 480, total scan time = 8 min.

Due to the relatively lower sensitivity of rodent imaging versus human imaging, to calculate a statistical map from phMRI we used a method based on Sanganahalli et al.^36^ Only data within the brain and not within cerebrospinal fluid were analyzed. For the first-level analysis, for each mouse separately, on a per-voxel basis, each fMRI scan was subdivided into 20 sections of length of 24 samples. All 24 samples at 0 min were taken compared to all 24 samples from either 30 or 60 min with a student’s *t* test (two-sample, equal variance).^36^ This produced 20 volumes per comparison (either 0 vs 30 min or 0 vs 60 min) of T maps for each mouse at level 1. For the second-level analysis, on a per-voxel basis, all 20 sections combined across all mice given vehicle and compared to all 20 sections combined across all mice at given rimonabant with a student’s *t* test (two-sample, equal variance). (24 volumes in the first level and 20 sections in the second level were chosen to approximately balance the first and second levels, as 24 × 20 = 480.) *p* values were corrected (separately per comparison, either 0 vs 30 min or 0 vs 60 min) by SGoF to correct the threshold of *p* < 0.05 for multiple comparisons.^86^ This produced a second-level map of significant brain regions for rimonabant vs vehicle, at either 0 vs 30 min or 0 vs 60 min.

### Statistical Analysis

A one-way analysis of variance (ANOVA) was performed, examining the effects of two variables on the metrics derived from DTI, fMRI, and STCT data. The first variable involved a comparison between genotypes (genotype versus wild type) or drug injection conditions (Rimonabant injection versus vehicle injection). The second variable involved a comparison between sexes (male versus female). The metrics upon which significance testing was conducted were two variance-based metrics (ALFF and fALFF), two FCD-based metrics (lFCD and gFCD) from fMRI data, and four variance-based metrics (FA, AD, RD, and MD) from DTI and STCT. The *p*-values were corrected based on the sequential goodness of fit metatest (SGoF) for family wise error rate (FWER).^86^

To compare specific brain regions that were significant for the interaction factor for STCT metrics in the previously described ANOVA test, we used Tukey’s honestly significant difference procedure (MATLAB function “multcompare.m”) to test for significance between the 4 groups of either male and female, either *cnr1*^*–/–*^ or WT mice.

## Supporting Information Available

Independent components resulting from ICA analysis; mean Pearson correlation coefficient (r-score) matrix of cnr1–/– mice and WT mice; rimonabant group and vehicle group; group statistical parametric map at 30 min time point; comparison of STCT between rimonabant and vehicle group; CB1 distribution in the brain; and abbreviations of brain regions (PDF)
