# Supplementary material for: Resting State Brain Networks under Inverse Agonist versus Complete Knockout of the Cannabinoid Receptor 1
Source: ACS Chem Neurosci. 2024 Apr 4;15(8):1669–83. doi: 10.1021/acschemneuro.3c00804 (PMC11027912; doi:10.1021/acschemneuro.3c00804)
Supplement: Supplementary file 1 — cn3c00804_si_001.pdf [file cn3c00804_si_001.pdf]

Supporting Information

Resting State Brain Networks Under Inverse Agonist versus Complete Knockout of the Cannabinoid Receptor 1

Hui Li[1, #], Qiong Ye[2, #], Da Wang[1,3], Bowen Shi[1,3] Wenjing Xu[4,5], Shuning Zhang[1,3], Xiaoyang Han[4,5], Xiao-Yong Zhang[4,5,*], Garth J. Thompson[1,*]

Affiliations

1. iHuman Institute, ShanghaiTech University, Shanghai, 201210, China.

2. High Magnetic Field Laboratory, Hefei Institutes of Physical Science, Chinese Academy of Sciences, Hefei, Anhui, 230031, China

3. School of Life Science and Technology, ShanghaiTech University, Shanghai, 201210, China.

4. Institute of Science and Technology for Brain-Inspired Intelligence, Fudan University, Shanghai, 200433, China.

5. Key Laboratory of Computational Neuroscience and Brain-Inspired Intelligence, Fudan University, Ministry of Education, Shanghai, 200433, China.

#These authors contributed equally

Contact information

* Address correspondence to:
gthompson@shanghaitech.edu.cn ShanghaiTech University, iHuman Institute, 393 Middle Huaxia Rd, Shanghai, 201210, China
Xiaoyong_zhang@fudan.edu.cn Fudan University, Zhangjiang Campus, 220 Handan Rd., Shanghai, 200433, China

Table of Contents

Figure S1. Independent components resulting from ICA analysis of BOLD-fMRI data for consideration as nuisance regressors.

Figure S2. Mean Pearson correlation coefficient (r-score) matrix of cnr1−/− mice and WT mice.

Figure S3. Group comparison of lFCD and gFCD values in individual brain regions

Figure S4. Group statistical parametric map 30 min after Rimonabant or vehicle injection.

Figure S5. Mean Pearson correlation coefficient (r-score) matrix of Rimonabant group and vehicle group.

Figure S6. Comparison of spatio-temporal correlation tensor between between Rimonabant and vehicle administration in mice

Figure S7. CB1 distribution in the brain.

Table S1. Brain regions used in main text, with abbreviations used in figures.

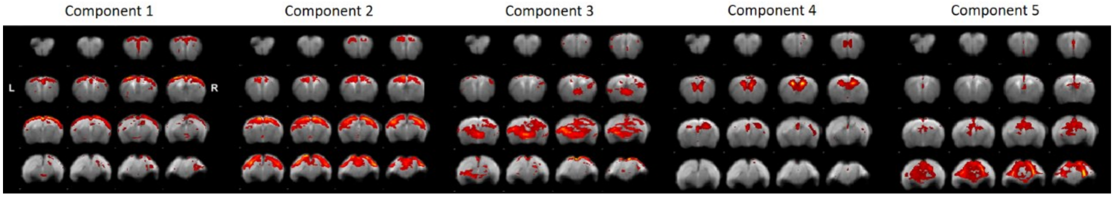

Fig S1. Independent components resulting from ICA analysis of BOLD-fMRI data for consideration as nuisance regressors. Each column represents one ICA component. The first 3 components appear to represent cortical and subcortical brain networks, whereas components 4 and 5 appear to represent ventricle and white matter signals and were removed. (N=12 per group.)

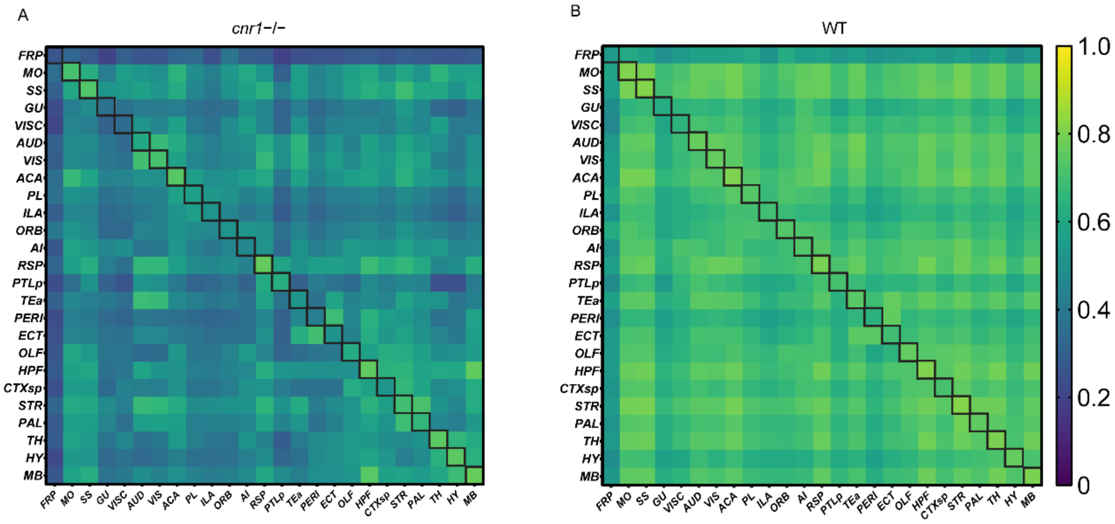

Fig S2. Mean Pearson correlation coefficient (r-score) matrix of *cnr1*$^{-/-}$ mice (A) and WT mice (B). (N = 10 for WT; N = 10 for *cnr1*$^{-/-}$) The lower left of the matrix represents the correlation coefficients between the ROIs of the left brain, the upper right represents the correlation coefficients between the ROIs of the right brain, and the diagonal represents the correlation coefficients between the left and right sides of the same ROI.

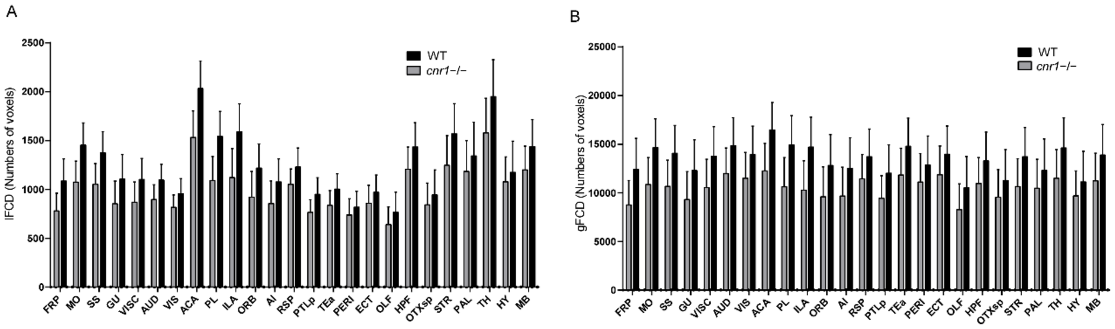

Fig S3. Group comparison of lFCD (A) and gFCD (B) values in individual brain regions. Data are mean ± s.e.m (N = 10 for *cnr1*$^{-/-}$ and N = 10 for WT).

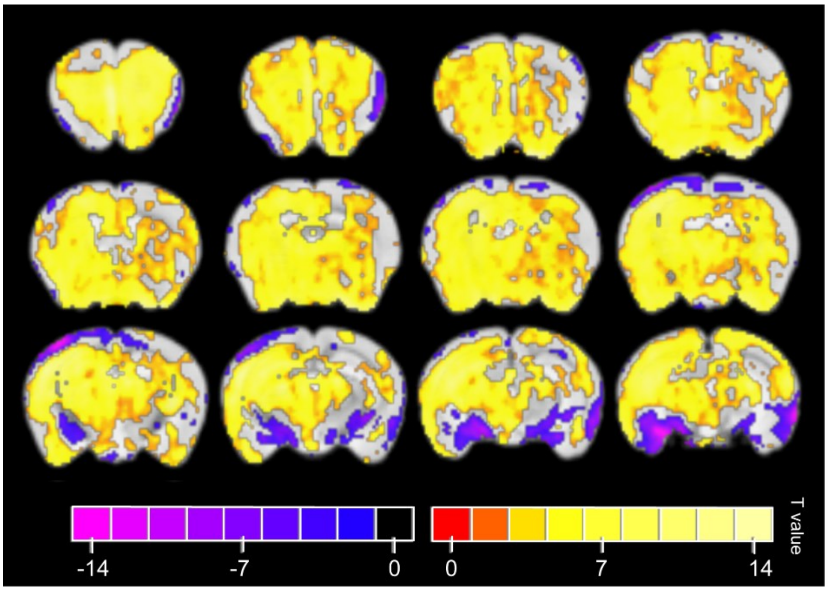

Fig S4. Group (n=8/group) statistical parametric map showing changes in BOLD contrast with significance threshold set to $p < 0.05$ (corrected with SGoF) following acute administration of Rimonabant (1 mg/kg body weight, i.p.), baseline vs. 30 min (first level), vehicle vs. Rimonabant (second level). Blobs in warm colors (left) indicate regions of increased BOLD signal compared with vehicle, whereas blobs in cool colors (right) are regions of decreased BOLD signal.

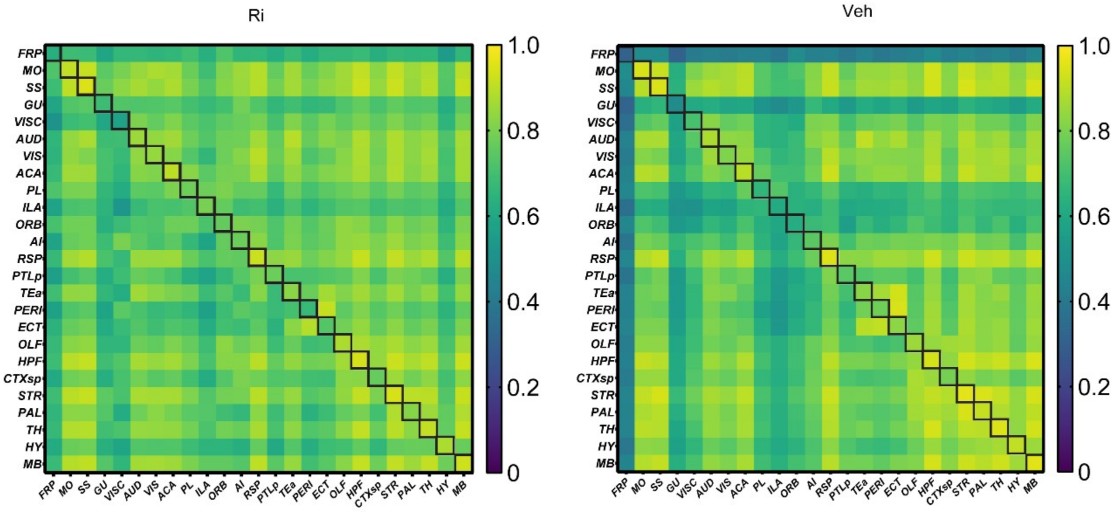

Fig S5. Mean Pearson correlation coefficient (r-score) matrix of Rimonabant group (A) and vehicle group (B) at 60 min. (N = 8 for Rimonabant group and N = 8 for vehicle group). The lower left of the matrix represents the correlation coefficients between the ROIs of the left brain, the upper right

represents the correlation coefficients between the ROIs of the right brain, and the diagonal represents the correlation coefficients between the left and right regions of the same ROI.

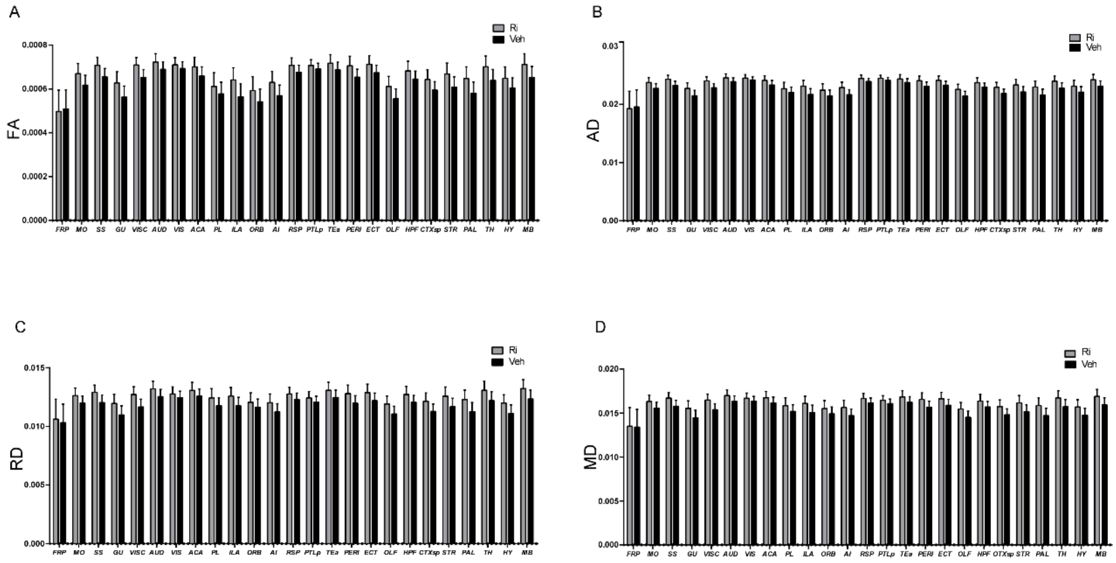

Fig S6. Comparison of spatio-temporal correlation tensor between Rimonabant and vehicle administration in mice. Group comparison of FA **(A)**, AD **(B)**, RD **(C)** and MD **(D)** in individual brain regions. Data are mean ± s.e.m (N = 8 for Rimonabant group and N = 8 for vehicle group).

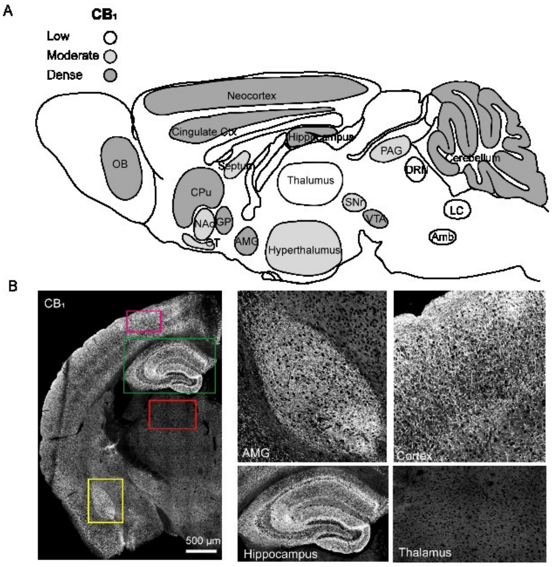

Fig S7. CB1 distribution in the brain. **(A)** Sagittal view of the mouse brain with several brain regions outlined, the inside of each brain region is gray-scale coded as shown above with the relative levels of CB1 in each region. **(B)** Immunostaining in the mouse brain showing relative CB1 distribution in amygdala (AMG), cortex, hippocampus, and thalamus. (N=1 WT mouse)

Table S1. Brain regions used in main text, with abbreviations used in figures.

Frontal pole	FRP
Somatomotor areas	MO
Somatosensory areas	SS
Gustatory areas	GU
Visceral area	VISC
Auditory areas	AUD
Visual areas	VIS
Prelimbic area	PL
Infralimbic area	ILA
Orbital areas	ORB
Perirhinal area	PERI
Anterior cingulate area	ACA
Agranular insular area	AI
Retrosplenial area	RSP
Posterior parietal association areas	PTLp
Temporal association areas	TEa
Ectorhinal area	ECT
Hippocampal formation	HPF
Olfactory areas	OLF
Cortical subplate	CTXsp
Striatum	STR
Pallidum	PAL
Thalamus	TH
Hypothalamus	HY
Midbrain	MB
Cranial nerves	cn
Cerebellum related fiber tracts	cbf
Lateral forebrain bundle system	lfbs
Extrapyramidal fiber systems	eps
Medial forebrain bundle system	mfbs
